# Fruits and vegetables at home (FLAM): a randomized controlled trial of the impact of fruits and vegetables vouchers in children from low-income families in an urban district of France

**DOI:** 10.1186/s12889-018-5908-5

**Published:** 2018-08-28

**Authors:** Camille Buscail, Aurore Margat, Stéphanie Petit, Judith Gendreau, Paul Daval, Pierre Lombrail, Serge Hercberg, Paule Latino-Martel, Aurélie Maurice, Chantal Julia

**Affiliations:** 1 Equipe de Recherche en Epidémiologie Nutritionnelle (EREN), Université Paris 13, INSERM U1153, Inra U1125, Cnam, Centre de Recherche en Epidémiologie et Biostatistiques (CRESS) Sorbonne Paris Cité, Bâtiment SMBH - 74 rue Marcel Cachin, F-93017 Bobigny cedex, France; 2 Département de Santé Publique, Hôpital Avicenne (APHP), 125 rue de Stalingrad, F-93000 Bobigny, France; 3 Laboratoire Educations et Pratiques en Santé (LEPS) EA3412, Université Paris 13, Sorbonne Paris Cité, Campus Condorcet, 74 rue Marcel Cachin, F-93017 Bobigny cedex, France; 4 Maison de la Santé de Saint-Denis, 6 rue des Boucheries, F-93200 Saint-Denis, France

**Keywords:** Fruits and vegetables, Food vouchers, Disadvantaged populations, Dietary behaviour

## Abstract

**Background:**

Fruits and Vegetables (FV) consumption is considered a marker of social inequalities in health since it is considerably decreased in disadvantaged populations. The main objective of this trial was to evaluate the impact of vouchers for FV purchase on the consumption of FV among children living in disadvantaged families in a French urban district.

**Methods:**

The FLAM study was a controlled randomized intervention trial, performed in Saint-Denis (North suburbs of Paris). The study group (intervention or control) was randomly attributed to parent-child pairs at inclusion. The intervention group received vouchers exchangeable for FV over a 1 year period. Nutritional education through workshops was available for both groups. FV consumption was assessed through face-to-face food frequency questionnaires. Participants who reported eating less than 3.5 FV per day were considered low FV consumers.

**Results:**

A total of 92 parent-child pairs were included, in which 45 were allocated to the intervention group and 47 to the control group. Amongst them, 64 completed the final follow-up questionnaire (30% lost to follow-up). After one year, the proportion of low FV consumers in children was significantly lower in the intervention group (29.4%) compared to the control group (66.7%, *p* = 0.005). Overall, 82% of the vouchers were used by the families.

**Conclusions:**

This study found a decreased proportion of small consumers in children after 1 year of distribution of FV vouchers compared to the control group. FV vouchers could be an effective lever to increase FV consumption among children from disadvantaged households.

**Trial registration:**

ClinicalTrials.gov identifier no. NCT02461238.

## Background

A sufficient fruits and vegetables (FV) consumption contributes to reducing the risk of non-communicable diseases, including obesity, type 2 diabetes, cardiovascular diseases (CVD) and several cancers [1–7]. Conversely, an insufficient consumption of FV has been estimated to be responsible for 19% of gastrointestinal cancers, 30% of ischemic heart diseases and 11% of strokes worldwide [3]. It is therefore considered as one of the top 10 factors leading to mortality worldwide, including in developed countries in which inadequate nutrition has become one of the leading mortality risk factors [3]. The world health organization (WHO) and the food and agriculture organization of the united nations (FAO) therefore recommend the consumption of at least 400 g of FV per day [8, 9]. In France, this recommendation has been translated as follows: people are encouraged to eat at least 5 servings of FV (i.e. of 80 g each) per day [10]. Low consumers of FV are defined as such when consuming less than 3.5 servings of FV per day [10].

Studies over the past two decades have provided important information on the association between socioeconomic status and dietary habits [11–18]. Overall, lower socioeconomic populations use to have a less healthy diet, and have higher proportions of small FV consumers in Northern Europe [15, 19, 20], United States of America (USA) [21], United Kingdom [22] and Australia [23]. In France, 35% of adults from the general population eat less than 3.5 servings of FV per day, this rate rises to 82.4% among underprivileged people using food aid [24–26]. This trend is also reflected in children, as those from lower socioeconomic groups consume an average of 2.6 to 3.0 servings of FV per day, whereas in general population, FV consumption is ranging from 3.4 to 3.6 servings per day in children [27]. Promoting healthy dietary habits among the youngest populations is an important issue since concrete food choices and dietary behaviors seems to originate in childhood and adolescence [28–31].

A study performed in the USA among subjects from the Supplemental Nutrition Assistance Program (SNAP) (*N* = 10,000), showed that a 30% increase in the FV budget decreased the cost of chronic diseases such as type 2 diabetes, CVD, strokes and obesity [32]. The financial cost of such assistance at the national level has therefore been estimated as a major public health benefit [32]. Several studies have documented the efficacy of FV vouchers on daily consumption among pregnant women and young children up to 5 from “nutritional risk” populations, like in the USA [33, 34] or in Great-Britain [35]. The findings showed that vouchers were associated with an increased consumption of FV, with a higher consumption when combining nutritional education [34, 36–40].

But so far, no such intervention was specifically conducted among children, most of studies being performed at school. [41, 42]. Yet, there is a real interest in promoting FV consumption simultaneously at school and at home, since it has been shown that dietary behaviours in these two environments are strongly associated [34, 43, 44]. Additionally, results from such studies performed among disadvantaged populations are often limited due to recruitment and follow-up difficulties [41]. The French National Nutrition and Health Program (*Programme National Nutrition Santé)* (PNNS) is a national public health program aiming to improve the health of the general population through nutrition. Two major aims have been included in this program: 1) decreasing the number of individuals considered as low consumers of FV (less than 3.5 servings per day) [45] by at least 25%, and 2) improving the nutritional status of disadvantaged population [2].

In this overall context, the “Fruits and vegetables at home” study (*Fruits et légumes à la maison*) (FLAM study) aimed at assessing the effect of FV vouchers on the daily consumption of FV in children from low-income families. The present study relied on a mixed research method aiming to 1) determine whether children from disadvantaged households receiving FV vouchers during one year modified their FV consumption (quantitative method), and 2) understand how the intervention impacted dietary practices and identify barriers and levers for participation through interviews of families (qualitative method).

## Methods

As stated above, this study was based on two complementary approaches. For clarity, method and results sections are divided in two distinct parts for each type of analysis.

### Quantitative analysis

#### Study design

The study was set in Saint-Denis city (Seine-Saint-Denis county, Ile-de-France region, France), as a location representative of disadvantaged urban areas in France. The unemployment rate is high (23.8% of unemployed in 2014 vs. 9.9% in France) [46], as well as the poverty rate (38.7% vs 14.1% in France) [46–49]. In 2012 in the county of Seine Saint-Denis, single-parent families represented 28.0% of families (versus 22.7% in France) [50, 51]. In addition, the prevalence of diabetes for all ages was 5.42% in Seine-Saint-Denis (while it is 4.82% in France) [52], and the obesity rate was 13.9% in children aged 5 years old vs 10.6% in mainland France [53].

**Fig. 1:**
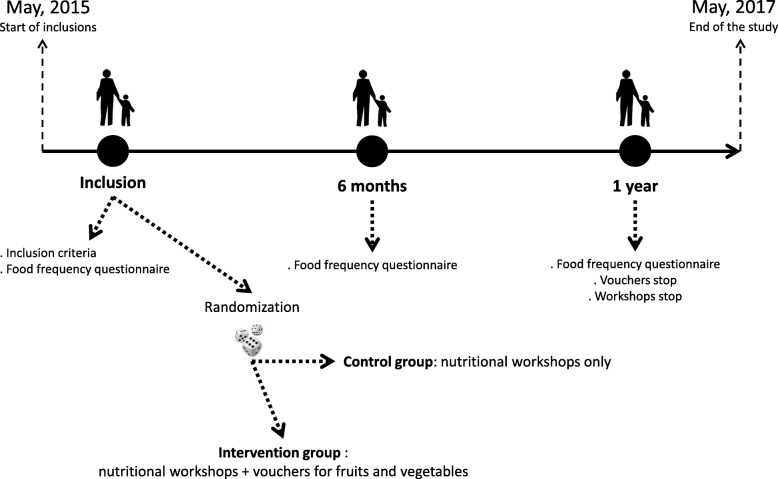
Description of the FLAM study

The FLAM study is a randomized trial assessing the effect of FV vouchers in low-income families in Saint Denis on FV consumption in children. Dietary assessments were performed at baseline, 6 months and 1 year. Both the control and intervention groups received nutritional education support through dedicated group workshops (Fig. 1). Participants were considered low FV consumers when they reported eating FV less than 3.5 times a day [45]. The primary outcome of the study was the proportion of low FV consumers among children at the end of the study. The secondary endpoint was the FV consumption among adults. Another secondary objective of the study was to assess the whole dietary behavior of participants at the end of the study.

#### Ethics

Each adult participant (whether the mother or the father included with his/her child) signed a consent form, after the interviewer made sure it was well understood. The study was approved by the Ethics Review Committee of the National Institute of Health and Medical Research (*Institut National de la Santé et de la Recherche Médicale*) (Inserm) IRB00003888 under the number 15–247. The declaration to the National Commission of Data Processing and Liberties (*Commission Nationale de l’Informatique et des Libertés*) (CNIL) of February 26 2015 was made under number 1838429v0. The study protocol has been registered on clinical trials website under no. NCT02461238.

#### Study population

##### Recruitment method

Recruitment was performed with the support of municipal services in the city of Saint-Denis, such as social workers, local associations and municipal health centres. Moreover, a large communication campaign before the implementation of the study was provided through posters, flyers and information in several community centres from January to July 2015. Permanencies to promote the study were held during neighbourhood festivals. Finally, a specific mailing of eligible participants was performed, using available information from the Saint-Denis family allowances fund towards target families.

##### Inclusion criteria

The target population was defined as follows: families with at least one child aged from 3 to 10 years old, living in the northern neighbourhoods of Saint Denis. Single-parent families were included exclusively from June 2015 to March 2016, then both single parent and couples were included until the recruitment period ended in May 2016. In addition participants had to have incomes below the poverty line, or receive social minima (Active Solidarity Income, Allocation of minimum pension), unemployment, and/or any income-terms allowance.

The poverty line threshold was defined after the French National Statistical Institute (*INSEE*) according to the French incomes data [54]. The first threshold was fixed at 1.234 euros per month rounded up to 1.300 euros for a single-parent household with at least one child aged under 14 years old. For a couple with at least one child aged under 14 years old the threshold of 1.777 euros per month was rounded to 2.000 euros [54]. Finally, French language had to be well spoken and understood.

#### Questionnaires and data collection

We included parent-child pairs. Data were collected via face-to-face questionnaires administered by trained interviewers at inclusion, then at 6 months and one year. Volunteer families were interviewed at community centers, or at home in order to sign the consent form, and complete the questionnaires. Questionnaires were adapted from those used in the ABENA study, which was specifically designed to be administered to disadvantaged groups [26]. A food frequency questionnaire was used to describe the consumption of children and adults in 13 main food groups (cereal products, starches, vegetables, fruits, legumes, dairy products, meats and eggs, fish and sea-food products, fast-food and pizza, salty snacks, sweet products, and beverages). This allowed assessing of the frequency of their specific daily, weekly or monthly consumption in each group. We relied upon French dietary guidelines to define frequency categories of all food groups in our population (see supplemental material) [10]. The questionnaire did not allow for an assessment of the quantity consumed in each occasion, and therefore the assumption was made that each eating occasion corresponded to a portion. The baseline questionnaire also included information on inclusion criteria, food supply, knowledge on French nutritional recommendations (assessed through 8 questions on consumption frequencies of various food groups and drinks), living conditions, and food security. The intervention group was also asked about the frequency use of the vouchers and behavioural changes regarding FV consumption in the Follow-up questionnaires, through the 4 following questions: 1) “In your opinion, has your consumption of FV increased over the past 12 months? ” 2) “If you didn’t use the FV vouchers, can you tell us why? ” 3) “You did use the vouchers, but did not increase your consumption of FV. Can you tell us why? ” 4) “Have you modified your behaviour regarding FV purchases? ” (purchases sites and types of FV). Every households received a gift coupon worth 10 euros after the interview.

#### Description of the intervention

##### Vouchers

At the end of the interview, parent-child pairs were randomly allocated in the intervention or control group through an algorithm of random distribution performed on a laptop. The algorithm was computed to balance groups every 50 inclusions. Families from the intervention group received vouchers (each voucher worth 3 euros), that could be used to buy fresh, canned or frozen FV, as well as 100% fruit juices. The vouchers were sent at household’s home by postal service every month during a year, and could be used in large and medium-sized supermarkets and several farmers’ markets in Saint-Denis city. The vouchers total amount fulfilled the cost of one serving of FV (equivalent 80 g) per day, for each member of the family. We relied on the average price of one kilogram of FV in France in 2014 (2.745 €) to estimate the allocation amount [55]. Thus, the number of vouchers sent to one household was consistent throughout the study but differed from one household to another depending on its size. A 2 people household received 4 vouchers (i.e. the equivalent of 12 €) every month, while a 3 people household received 6 vouchers (i.e. the equivalent of 18 €). Households of 4 people and more received 8 vouchers (i.e. the equivalent of 24 €) a month. The vouchers were electronically traceable to each household thanks to a unique barcode scanned at supermarket checkout. We were therefore able to keep track of the vouchers use in each family from the intervention group.

##### Workshops

All families, regardless of their group were invited to participate in the nutritional education workshops. These latter were prepared and led by one of the two dieticians of the study at a rate of around once a month, in two community centre of Saint-Denis. A part of the workshops was theoretical and aimed at increasing the knowledge on diet and specifically on FV through exchange and question sessions with the dieticians. The following are some examples of topics that were proposed: “balanced diet with small budget”, “Dealing with neophobic behaviour of children”, “What are the various food groups (for children)”. Otherwise, cooking workshops were proposed, to bring new culinary skills and recipes based on FV. Durations of workshops could vary from one to three hours, depending on the topic and/or the number of persons attending.

Families were informed by phone (SMS) about workshops which took place at the closest community centre from their home place. Contact with families, inscriptions and actual participation to the workshops have been collected.

#### Statistics

##### Sample size calculation

Sample size computation took into account a type I error of 5% and with an expected power of 90%. The baseline proportion of low consumers was expected to be the same as the ABENA study, 83.9%. The target proportion of low consumers was expected equalling those of the general population, 61.0%. This led to an expected number of participants of 92 in each group, leading to a total of 184 participants. The percentage of people lost to follow-up was estimated to be about 40%, leading to an expected number of participants of 300 [56].

Control group and intervention were compared according to their baseline characteristics. Quantitative variables were compared using Wilcoxon-Mann-Whitney tests and qualitative variables were compared using Fisher’s exact tests.

The proportion of low FV consumers in children (primary endpoint) and adults (secondary endpoint) between the intervention and control groups one year after inclusion were compared using Fisher’s exact test. FV consumer categories were established based on the food frequency questionnaire. Participants (adult or children) consuming FV less than 3.5 times a day were considered low FV consumers. Dietary intakes between intervention and control groups were compared according to the French dietary guidelines boundaries [10]. End-of-study consumption frequencies were then compared between control and intervention groups using Fisher tests. We first ran a per protocol analysis, based solely on the households which were followed throughout the study. Then, we performed an intention-to-treat analysis for the FV consumption, by replacing missing data on primary and secondary endpoints (i.e. FV consumption status in children and adults respectively) using a regression multiple imputation method (proc mianalyze of SAS software) [57–62]. Missing data were estimated conditionally on the following variables: child’s gender, adult’s gender, child’s age, adult’s age, size of the family, income level, professional status, marital status, Epices deprivation score, parent’s place of birth, food insecurity status, study group and FV consumption status at inclusion in the both parent and child. Twenty data sets were generated from which a pooled analysis (univariate logistic regression model) was then performed. Participants lost to follow-up were compared to those retained in the study after one year follow-up, according to the main characteristics at inclusion.

All statistical analyses were two-tailed and the type I error was set at 5%. All analyses were performed using SAS software (*version 9.4, SAS Institute, Cary, NC, USA*) [63], except for power calculations which were performed using the G* power software [64].

### Qualitative analysis

The qualitative analysis had two main objectives: 1) explore how FV vouchers were used by the households and question their participation in nutritional workshops, 2) understand how the program modified dietary practices and nutritional behaviours over the long-term. The qualitative survey was conducted from March to September 2017. It was performed through semi-directive face-to-face interviews, most often at home, among families from the intervention group. Participants were recruited on a voluntary basis. The number of interviews was determined using the data saturation method, based on the assumption that new collected data do not bring any new element to the understanding of the phenomenon under study. The data were collected by audio recording before being transcribed and anonymized. All interviews were analysed through thematic content analysis. For each of them, a first intuitive reading, called “floating” reading, allowed the emergence of the main ideas of the speeches [65]. A second reading called “in-depth” was done, focusing on the meaning of the discourses [66]. The analysis identified on the one hand units of meaning called “functional units”, of varying sizes, and on the other hand the “nucleus of meaning” corresponding to the different units of meaning [67, 68]. This allowed us to categorize the results into: headings/Subheadings - Themes - Sub-themes - Sub-themes - Units of meaning. The categorization of the data was done using an inductive approach allowing us to establish a typology of the subjects, according to their attitudes and behaviours related to the program.

## Results

### Quantitative analysis

#### Study sample

**Fig. 2:**
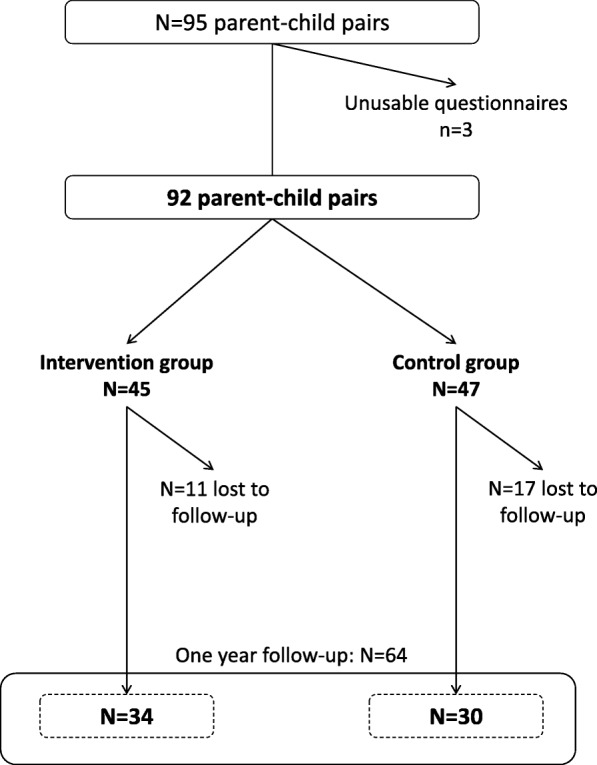
Flowchart of the FLAM study

From June 2015 to May 2016, 95 families were included in the study. Given we faced to recruitment difficulties, and although the recruitment period has been extended (from 6 month initially to 12 months), inclusions were stopped before 300 subjects. Three participants were excluded because of missing data on FV consumption. Finally, 92 subjects were included (Fig. 2). Follow-up questionnaires and workshops ended in May 2017 (i.e. one year after the last families were recruited).

Children aged from 6 to 10 years were more represented than younger children, with a majority of girls. Most of them had lunch at the school canteen. The included adults were mostly women, aged from 21 to 57 years, single-parent with up to 6 children. Most of the included adults (67.4%) were born in a foreign country, mainly located in Maghreb and Sub-Saharan Africa (*N* = 29.3% for each location). Two third (64.1%) of children were considered low consumers of FV as well as 78% of adults. Except for the children age, control and intervention groups did not differ on sociodemographic characteristics at inclusion (Table 1).

**Table 1:** Sociodemographic characteristics of households at inclusion according to the group (*N* = 92)

	Control group *N* = 47		Intervention group *N* = 45		*p* value*
	N	%	N	%	
Child female gender	27	57.4	24	53.3	0.69
Child age (years) (mean+/-SD)	6.8	±2.4	8.1	±2.2	0.01
Adult female gender	47	100	44	98	0.3
Adult age (years) (mean+/-SD)	39.3	±8.2	39.8	±6.4	0.72
Single parent household	44	93.6	41	91.1	0.65
Parent’s place of birth					
France^†^	18	38.3	12	26.7	0.53
Maghreb	11	23.4	16	35.6	
Sub-Saharan Africa	14	29.8	13	28,9	
Other	4	8.5	4	8.9	
Total number of children in household					
1	14	29.8	12	26.7	0.84
2	18	38.3	16	35.6	
≥ 3	15	31.9	17	37.8	
Household’s monthly income (euros) (*N* = 91)					
< 900	13	27.7	16	35.6	0.57
[900–1300[	28	59.6	25	55.6	
> 1300	5	10.6	4	8.9	
Small FV consumers (< 3.5 servings per day)					
Children	31	66.0	28	62.2	0.71
Adults	36	76.6	36	80.0	0.69
Lunch at school	39	86.7	40	88.9	0.75
EPICES Score (mean+/-SD)	54.1	16.1	61.1	16.9	0.05
Proportion of total food budget devoted to FV					
< 30%	28	59.6	22	48.9	0.3
≥ 30%	19	40.4	23	51.1	

Abbreviations: EPICES: Deprivation score ranking from 0 (the less precarious situation) to 100 (the most precarious situation). Precarious situation is defined when EPICES score is upper than 30.17, and great precarious situation when the score is upper than 53.84; FV: fruits and vegetables;SD: Standard Deviation;

^*^Fisher exact tests were performed for qualitative data and Wilcoxon-Mann-Whitney tests were performed for quantitative data

^†^Including Metropolitan France and overseas departments

Missing data: Proportion of food budget devoted to FV *N* = 5 (5.4%), other missing data were less than 5%

One year after inclusion, 64 families responded to the last survey, including 34 households in the “intervention” group and 30 in the “control” group (53.1 and 46.9%, respectively). Among these 64 households, 8 did not respond to the 6-month questionnaire. A total of *N* = 28 families (30.4%) were lost to follow-up. One participant was excluded from study because of relocation during the follow-up period.

#### Fruit and vegetable consumption

One year after inclusion, the proportion of children considered as low FV consumers in the intervention group was significantly lower than in the control group (29.4 95% CI [14.1–44.7] vs. 66.7 95% CI [49.9–83.5], *p* value = 0.005). This difference was not significant in adults (61.8 95% CI [45.5–78.1] and 76.7 95% CI [61.6–91.8] respectively in the intervention group and in the control group *p* = 0.28) (Table 2). The effect sizes were estimated at 37.3% for children and 14.9% for adults.

**Table 2:** Frequency of food servings in children at 1 year follow-up according to French dietary guidelines (*N* = 64)

	Total		Intervention group (*n* = 34)		Control group (*n* = 30)		p*
	n	%	n	%	n	%	
Fruits and vegetables (per day)							
< 3.5	30	46.9	10	29.4	20	66.7	0.005
≥ 3.5	34	53.1	24	70.6	10	33.3	
Dairy products (per day)							
< 3	27	42.2	12	35.3	15	50.0	0.31
≥3	37	57.8	22	64.7	15	50.0	
Fatty and salty products (per week)							
< 1	7	10.9	4	11.7	3	10.0	0.51
1 or 2	30	46.9	17	50.0	13	43.3	
> 2	27	42.2	13	38.3	14	46.7	
Sweet products (per week)							
≤1	8	12.5	3	8.8	5	16.7	0.72
[2;5[	5	7.8	2	5.9	3	10.0	
≥5	51	79.7	29	85.3	22	73.3	
Fish and seafood products (per week)							
< 1	28	43.8	14	41.2	14	46.7	0.72
1 or 2	29	45.3	17	50.0	12	40.0	
> 2	7	10.9	3	8.8	4	13.3	
Starchy food (per day)							
< 3	16	25.0	9	26.5	7	23.3	1
=3	2	3.1	1	2.9	1	3.3	
≥ 3	46	71.9	24	70.6	14	73.4	
Meat, fish and eggs (per day)							
< 1	11	17.2	3	8.8	8	26.7	0.15
1 or 2	32	50.0	20	58.8	12	40.0	
> 2	21	3.8	11	32.4	10	33.3	

*Fisher exact test

Consumption of FV (i.e. number of servings per day, using median (min-max)) was also compared between the two study groups as a continuous variable, using a Wilcoxon-Mann-Whitney test. One year after inclusion, daily consumptions of FV were significantly higher in the intervention group for both children (4.0 servings (1.4–6.0) compared to 2.2 (0.9–5.0) with *p* value< 0.001) and adults (3.0 servings (0.5–7.0) compared to 1.9 servings (0.1–5.0) with p value = 0.02) (data not tabulated).

#### Secondary criteria

##### Consumption according to the French dietary guidelines

Except for FV consumption, food group consumption (categorized from the French Nutritional guidelines) at the end of the trial did not significantly differ according to the allocation group. Children consumption patterns were similar to those of parents, except for the dairy products, for which more than 50% followed the recommendations, while only 7% of parents did (Table 3).

**Table 3:** Frequency of food servings in adults at 1 year follow-up according to French nutritional guidelines (*N* = 64)

	Total		Intervention group		Control group		p*
	n	%	n	%	n	%	
Fruits and vegetables (per day)							
< 3,5	44	68.7	21	61.8	23	76.7	0.28
≥ 3,5	20	31.3	13	38.2	7	23.3	
Dairy products (per day)							
< 3	60	93.7	32	94.1	28	93.3	0.17
≥3	4	6.3	2	5.9	2	6.7	
Fatty and salty products (per week)							
< 1	15	23.4	10	29.4	5	16.7	0.64
1 or 2	38	59.4	20	58.8	18	60.0	
> 2	11	17.2	4	11.8	7	23.3	
Sweet products (per week)							
≤1	35	54.7	18	52.9	17	56.7	0.36
[2;5[	16	25.0	10	29.4	6	20.0	
≥5	13	20.3	6	17.7	7	23.3	
Fish and seafood products (per week)							
< 2	39	60.9	22	64.7	17	56.7	0.76
2 or 3	22	34.4	11	32.4	11	36.7	
> 3	3	4.7	1	2.9	2	6.6	
Starchy food (per day)							
< 3	27	42.8	12	35.3	15	51.7	0.31
3	1	1.6	1	2.9	0	0	
≥ 3	3	55.6	21	61.8	14	48.3	
Meat, fish and eggs (per day)							
< 1	17	26.9	10	29.4	7	24.1	0.21
1 or 2	27	42.9	17	50.0	10	34.5	
> 2	19	30.2	7	20.6	12	41.4	

*Fisher’s exact test

##### Use of vouchers (intervention group)

Out of 3614 vouchers sent to families in the intervention group, 2928 vouchers (81%) were used from the 07th September 2015 to the 31st October 2017. Most of families (*N* = 31 of 34 (91.2%)) reported that vouchers helped them for increasing their FV purchases and consumption. Only 3 households reported they used the vouchers without increasing their FV consumption.

##### Participation to workshops

A total of 30 sessions were proposed from September 2015 to June 2017. About 50 % of families (*N* = 49 among the 95 families initially included and therefore allowed to participate) came to at least one workshop. The participation rate (i.e. the proportion of families who came to at least one workshop within each study group) was slightly higher in the intervention group (56.5%) compared to the control group (46.9%), but not significant (*p* = 0.41, Fisher exact test, data not tabulated). There was no association between workshop attendance and changes in FV consumption in children (*p* = 0.79) neither in adults (*p* = 0.58) (data not tabulated). Children who were small consumers at one year were as well represented (50%) as non-small consumers among children who went at least once to a workshop.

##### “Intention-to-treat” analysis

After multiple imputation, proportions of children consuming less than 3.5 FV per day were 26.6% in the intervention group and 64.2% in the control group. These proportions were 63.4 and 76.6% in adults, respectively. Differences between intervention and control groups resulting from the pooled analysis showed similar *p*-values as the per-protocol analysis: 0.003 in children and 0.24 in adults. Comparisons between families lost to follow-up and other families showed no significant difference, except for the Epices score (assessing precarious situation), which was higher in families lost to follow-up (64.4 +/− 15.7 vs 54.5 +/− 16.5 for families who remained in the study after one year, *p* = 0.01) (Table 4).

**Table 4:** Comparison of the main characteristics (at inclusion) between families lost to follow-up and those maintained in the study after one year

	Families followed over one year		Families lost to follow-up		
	*N* = 64		*N* = 28		
	N	%	N	%	p*
Parental age (mean ± std)	39.6	±7.3	39.5	±7.6	0.97
Child’s age (mean ± std)	7.4	±2.5	7.6	±2.1	0.91
Country of birth					
France	21	32.8	9	32.1	0.95
Other	43	67.2	19	67.9	
Marital status					
Single	58	90.6	27	96.4	0.67
Cohabiting	6	9.4	1	3.6	
Education level					
Primary school	17	26.6	12	42.9	0.35
Secondary school	25	39.1	10	35.7	
College	20	31.2	5	17.9	
Other	2	3.1	1	3.6	
Occupational status					
Unemployed	45	70.3	21	75	0.64
Working	19	29.7	7	25	
Income level					
900 € per month	17	27.0	5	17.9	0.06
900–1300 € per month	24	38.1	18	64.3	
> 1300 € per month	22	34.9	5	17.9	
Epices score (mean ± std)	54.5	±16.5	64.4	±15.7	0.01
Perception of the financial situation					
« It’s ok »	2	3.1	0	0	0.56
« I need to be very careful »	24	37.5	8	28.6	
« It’s difficult »	23	35.9	10	35.7	
« I can’t make it without debts »	15	23.4	10	35.7	
Food insecurity					
Secure	23	35.9	6	22.2	0.43
Food insecurity without hunger	16	25	8	29.6	
Food insecurity with hunger	25	39.1	13	48.1	
Have used food aid over the last 12 months					
Yes	16	25.8	6	21.4	0.65
No	46	74.2	22	78.6	
Number of child in the household					
1	14	21.9	8	28.6	0.80
2	17	26.6	7	25	
≥ 3	33	54.6	13	46.4	

*Fisher exact tests for all variables except for parental age and Epices score: Student test and child’s age: Wilcoxon-Mann-Whitney test; Abbreviations: std. standard deviation; € euros; Missing data: Epices score: *n* = 5 (5,4%);

### Qualitative analysis

Thirteen women participated to the qualitative survey. Twelve out of 13 were born abroad: North Africa (5), sub-Saharan Africa (4), Moldova (1), Haiti (1) and the Comoros (1). All of them were single, with one or more children (maximum of 4). They received information about the program through: a letter (3), the municipality of St Denis (3), the neighbourhood house (2), a neighbourhood festival (2) or a poster seen in the city (1). All of the participants reported they used “all” or “almost all” of the vouchers when the interview was performed. Four headings were identified, leading to the following categorization: respondents’ dietary habits; the FLAM program process; participants’ feelings; and the impact of the FLAM program. This first categorization led us to a typology based on: baseline participants’ knowledge on diet (primarily regarding FV that can be found in France, the way they can be prepared and consumed); their knowledge/adherence to PNNS (French Nutrition and Health National Program) guidelines; respondents’ dietary practices prior to their participation to the program; the ways they used the vouchers and their dietary habits during and after the program. Three profiles were identified after the categorization:

#### Participants with poor or no knowledge on FV (health interest, preparation) and who did not participate to workshops (4 participants of 13)

For these participants, the reason for participation was mainly financial: “I don’t work, so it was a help”; “It was to have some extra money... the vouchers helped to save money”. They used the vouchers for products they already knew and ate (fruit juices, compotes...) and used the saved money to buy other food items (mainly meat, starchy foods, milk, branded sweet products, fast food “reward” for children) and so on:“... *There are things I can’t usually buy and that I was able to buy thanks to the saved money: fish, meat. It helps*”; “*The vouchers helped me to save money... From time to time I was able to buy pizza...*”; “*I made a box with the money I was saving and the girls could use it to buy a sandwich from time to time*”. Although these women reported increasing their consumption of FV at the time of the program, no change was observed in long-term behaviour (after the program):“I eat the same way I used to do before the program)”.

#### Participants with little or no knowledge about FV (mode of preparation or association between consumption and health) and who participated to the workshops (5 participants of 13)

In this profile, the reasons for participation were both financial and improvement of their knowledge about FV (for instance, learning how to cook vegetables they were not used to). They used vouchers to increase their consumption of FV, mostly by buying new varieties they discovered during the workshops. Participants expressed a satisfaction related to the development of new knowledges/culinary skills: “*We learned a lot of things... I even made a cake with my son... It was really very good*”; “*I learned a lot. They gave a lot of recipes*”; “*The thing I liked the most was the interventions with the dietician, I learned a lot about new vegetables and fruits*.”. These women also shifted the budget towards other foods (meat, starchy foods, milk, branded sweet products, fast food “reward” for children) but also towards other products than food (such as housing): “*I was able to put money aside for the apartment*”. After the program ended, these participants continued to keep an increased daily FV consumption compared to those they used to have prior to the FLAM program: “*I realized that my daughter liked it... So I continue to buy them*”; “*Now for the snack I give my son a fruit or a compote*”.

#### Participants with a baseline knowledge about FV (i.E. understanding that FV are important for their health and they children’s’, and knowing the PNNS guidelines) (4 respondents/13).

The reason for participation was financial and for 2 of 4 participants, there was also an interest in contributing to this type of study in order to help society “to make a difference”: “*if it can help you, why not...*”. Participants of this profile were all from North Africa (4/4), coming from privileged backgrounds (3/4): “*I come from a middle-class family*” and/or had a higher education level (2/4). They were involved in the neighbourhoods’ life (community centre, parents’ associations...). They reported using the vouchers to buy FV they knew but could not usually purchase for financial reasons (for instance strawberries, raspberries, organic vegetables): “*I was able to buy them [the children] vegetables they like and that I don’t usually buy... like red berries*”. These women reported a significant increase in FV consumption along the program duration (in both quantitative and qualitative ways). The budget was also shifted towards other food items such as fish, meat and organic products (but not starch, milk, sweet products and fast food). Two women also participated in the workshops, intending to meet the professionals and share with the other participants. After the program ended, they recovered their previous FV consumption (the one they had before the program). Beyond these 3 profiles, all women reported some difficulties in using their vouchers, mostly because sellers were sometimes reluctant to accept them. First, most of the women interviewed (11 of 13) were used to buy their FV at the market, where vouchers were not widely useable. They therefore needed to change their food purchasing habits. In addition, it appeared that cashiers in partner superstores were insufficiently aware of the FLAM vouchers. This led to difficulties at the time of payment (refusal or intervention of the manager) which could result in a feeling of “embarrassment”, or “inconvenience” for women: “*The stores should be told that they are obliged to take the vouchers...*”; “*Several times, the cashiers called their superior*”. It should be noticed that even if almost all interviewees reported such type of difficulties, women belonging to group iii (see above) seemed less “embarrassed” by these issues, feeling more able to manage the situation: “*When they did not want to take the vouchers, I had the supervisor called*”.

Regarding participation in collective workshops with the dietician, one of the main difficulties related to the schedules which did not always match with women’s availabilities, in particular those who were working and/or were single: “*The schedules for workshops should be reviewed. I don’t have the time to participate”; “ I wasn’t working at the time, that’s why I was able to participate. Today, I could not”*.

## Discussion

This study showed a significant increase in FV consumption in children from disadvantaged families with a one-year allowance of FV vouchers compared to a control situation. No significant difference was observed in adults. Consumption of other food groups was not modified according to the study group.

Our results though being modest, remain encouraging and are in line with previous studies performed using similar interventions. An English study compared three adult groups, a “control” group (*N* = 64), a “nutritional advice on fruit and fruit juice” group (*N* = 63) and a “coupons for fruit or fruit juice” group (*N* = 63). Results showed that only the group receiving vouchers significantly increased fruit and fruit juice consumption compared to other groups [35]. In Dunedin, New Zealand, a randomized study of 151 volunteers, (*N* = 81 in the intervention group), showed a significant increase in overall food expenditure when coupons were distributed. However, the vouchers were not specifically targeting FV, but healthy foods items [69]. In the USA, studies in the national WIC program showed significant positive impacts of vouchers on FV consumption, especially when strengthened by nutritional education [34, 36, 38, 39]. In France, a randomized study on the effect of FV vouchers on consumption among adults was performed in the same area as FLAM study in 2008 [56]. Vouchers did not show significant positive effects on the mean consumption of FV after 6 months; but they significantly decreased the proportion of very small FV consumers (i.e. < 1 servings a day) [56]. Due to important attrition rates, results at 9 and 12 months attrition rates (respectively at 55.3 and 84.8%) were not available.

Non-randomized intervention studies were conducted in England, in the context of the Healthy Start program, in which vouchers were distributed to pregnant women and up to 4 years of age of the child. A total of 113 volunteers reported during the focus group that they increased quantitatively and qualitatively their FV consumption through coupons [70]. A second study, conducted over a 5-month period (*N* = 621), and using FV prescription by medical professionals did not show any significant effect on consumption, though the knowledge of the slogan “Five-a-day” was increased in the population [71]. More generally, a review performed in 2012 on the effectiveness of subsidies in promoting healthy food purchases and consumption concluded that these types of interventions tended to be effective in modifying dietary behavior [41].

Overall, these results tend to support the fact that vouchers alone may not be sufficient to increase consumption, and that the addition of nutrition education is suitable [72]. On the other hand, Darmon and colleagues have shown that a nutrition education appears inefficient, if not supported by a financially affordable supply of healthy food [73]. These findings are in line with our qualitative survey, showing that women with a baseline poor knowledge about FV who participated in the workshops durably modified their dietary habits. Furthermore, a financial support for the entire household is relevant (instead of children only), since parents are primary responsible for the food choices of the entire household, they also act as a model for their children [71]. Our results were consistent with this assumption since the assessment of the adherence to the PNNS dietary guidelines showed that children’s dietary profiles were overall similar to their parents’ (Table 2) [71, 74]. Furthermore, several works have highlighted the close interrelationship between FV consumption in parents and children, in both directions [71]. An evaluation study of the “5 a day” program in Los Angeles showed that when a mother increased her daily FV consumption, it had a positive impact on the consumption of entire household [37].

Surprisingly, no significant effect of the intervention on FV consumption has been shown in parents. However, the considerable use of vouchers suggested that the FV affordability in the households was increased. We assume that parents prioritized their children consumption, giving them the most of purchased FV, before raising their own consumption. A possible explanation for this might be that the number of vouchers sent did not always match with the size of the household (maximum 8 vouchers per month for households with 4 people or more).

A large majority of children (88%) in our study population had lunch at school canteens, in which the nutritional quality of foods is regulated in France [75–77]. The school catering of Saint-Denis showed a good compliance to these recommendations [78]. This may partly explain the significantly higher FV consumption (Exact Fischer test *p* = 0.001, data not shown) in children compared to adults at baseline. However, though school canteens appeared to partly increase children’s FV consumption, the amount provided did not appear to compensate for an overall low consumption (compared to the general population).

Given the results on our primary outcome after 1 year follow-up (29.4 and 66.7% of low FV consumers in the intervention and control group respectively), and the number of children within each group (*n* = 30 in the intervention group and *n* = 34 in the control group), the effect size was therefore estimated at 37.3% and the power of the study at 31.9% [79]. According to Cohens’ thresholds regarding effect sizes (0.20 is small and 0.50 is medium), the effect sizes of the intervention can be considered moderate in children (0.37), and small (0.15) in adults [79].This could partly explain why no significant result was seen in adults, despite a lower proportion of small FV consumers in the intervention group (61.8%) compared to the control group (76.7%) after one year follow-up (Table 3). On the other hand, the results observed in children are quite encouraging for this kind of intervention. Compared to the study of Bihan and colleagues, our effect size in adults was lower (respectively 20.3% vs 14.9%). Regarding the daily consumption of FV, Herman and colleagues found an effect size of + 3.0 servings per day between households from voucher and control groups, while this difference was at estimated + 1.8 servings in children and + 1.1 servings in adults in our study. Burr et al. showed that the purchase of healthier food increased by 6% during the intervention.

The attrition rate of 30.4% is much lower than those found in a similar study previously performed by Bihan and colleagues in the same area [56]. Moreover, it is in line with previous interventional researches performed among similar populations. Katz and colleagues performed in2001 an interventional study among low-income mothers with an attrition rate of 41% after one year duration [80]. Nicholson and colleagues described in 2011 several retention strategies they implemented to improve retention rates in their interventional research in a low-income urban population. Attrition rates were 25% et 36% at 6 and 12 months respectively [81]. Recruitment barriers have been explored through a qualitative analysis [82]. Briefly, the main reasons reported by the individuals who refused to participate in the study were a lack of time, mistrust towards researchers and people coming from outside the neighborhood, and trouble with communicating with the interviewers.

Our study had some limitations. First, we used a food frequency questionnaire to assess food consumptions instead of a 24-h dietary recall which is usually the gold-standard for dietary assessment. This could have led respondents to misestimating some food consumptions (in particular FV), mostly due to a memorization bias. Plus, this type of assessment does not allow assessing the portion sizes [83]. Moreover, due the young age of their child or its inability to respond to the questionnaire directly, some parents had to answer instead of their child (*N* = 14) which may have led to less reliable information. Besides, the frequency dietary questionnaire we relied on has been specifically designed to be administered to disadvantaged groups, and was previously used in the French ABENA study performed among food aid users [84, 85].

Another limitation pertains to the limited number of vouchers per household. Indeed, 34.8% of households included had at least three children. Thus, vouchers were not fulfilling the equivalent of one portion of FV a day for each person in these households. Sensitivity analyses in this group (*N* = 14) showed that there was no significant increase in FV consumption in these families. However, caution is needed when interpreting this result given the very low number of participants in this sub-group. We could not totally exclude a contamination, i.e. a transfer of a part of FV vouchers towards the families of the control group. Nevertheless, families were included and followed separately and few have indicated knowing each other. A contamination seems therefore unlikely. Finally, results showed that families lost to follow-up were more precarious than the others (Table 4). This finding is in line with the literature showing that daily life difficulties in such populations make them harder to reach and follow in trials [86]. We therefore explored the intervention effect by controlling the precariousness level of the families. In children from high level precarious families, difference in proportion of low FV consumers between the intervention and control group was lower than in other families, and was no longer significant (*p* = 0.16, data not tabulated). This should be kept in mind before extending the measure, in order not to worsen social inequalities, trying to reduce them.

The major strengths of this study were the randomized design of the intervention and the use of face to face interviews to collect the data. Moreover, the percentage of participants lost to follow - up at one year was less (30.4%) than expected, given the targeted population [56]. This could partly be due to regular solicitations of participants through follow-up questionnaire and workshops. The one-year duration of the FLAM study was another strength, a short duration being an usual limitation of such studies. It overcame the novelty effect of the vouchers and allowed them to be incorporated in usual purchase habits. Finally, the intention-to-treat analysis showed similar results, supporting the hypothesis of an efficiency of the intervention.

Finally, given the results of this interventional research, it is likely to consider its replication, or even its implementation at the national level (in the line with previous programs that were developed in the US or in the United Kingdom). However, maintaining this program in its current form in the long-term state seems difficult. Indeed, it would require significant human resources to handle nutritional education on such a large scale. Though, an adaptation of the financial incentives through a FV allowance for instance would be interesting to explore.

## Conclusion

The findings of this study, in line with similar researches conducted in other countries lead to two main conclusions: 1) vouchers for fruits and vegetables have been widely used by households from the intervention group and 2) children from the intervention group reported an increased FV consumption after one year follow-up. There is a strong scientific knowledge about the association between FV consumption and health and the social inequalities regarding their consumption in France. This study could therefore be a basis for the implementation of a wider public health policy planning for instance to allocate an amount dedicated to FV towards disadvantaged families.

## Abbreviations

95% CI: 95% Confidence Interval
CNIL: National Commission of Data Processing and Liberties
FAO: Food and Agriculture Organisation of the United Nations
FV: Fruits and Vegetables
INSEE: French National Statistical Institute
INSERM: National Institute of Health and Medical Research
PNNS: French National Nutrition and Health Program
SNAP: Supplemental Nutrition Assistance Program
WHO: World Health Organization
WIC: Women Infant Children
